# Neurology Residency Training in 2022: Insights from the American Academy of Neurology's Resident Survey

**DOI:** 10.1212/NE9.0000000000200221

**Published:** 2025-05-16

**Authors:** Brian E. Emmert, Abhishek Lenka, Jessica Frey, Laura A. Gilbert, Tasha Ostendorf, Carol E. Rheaume, Gauri V. Pawar

**Affiliations:** 1 Department of Neurology, Perelman School of Medicine, University of Pennsylvania, Philadelphia;; 2 Department of Neurology, Baylor College of Medicine, Houston, TX;; 3 Department of Neurology, West Virginia University, Morgantown, WV;; 4 Division of Neurology, Department of Pediatrics, Northwestern University Feinberg School of Medicine, Chicago, IL; and; 5 American Academy of Neurology, Minneapolis, MN.

The American Academy of Neurology (AAN) has been instrumental in identifying trends in neurology training in the United States by periodically surveying neurology residents and fellows regarding their training and perceived needs as they transition into practice.^1,2^ This survey assesses experiences in several domains, including fellowship choices, student loans, and business/practice management.^1,2^ The results of these surveys have been pivotal in identifying critical deficiencies within neurology training, which the Graduate Education Subcommittee and workgroups at the AAN systematically address. This report details the results of the current survey and identifies areas for future improvement.

A 30-item needs assessment survey was developed by AAN staff and survey workgroup based on the previously administered 2017 instrument and approved by the AAN Member Research Subcommittee. New items were added to address changes to residency training. The final survey queried postgraduate training, student loans, business management, research experience, clinical experience, fellowship time line, and subspecialty exposure. The survey was administered through Qualtrics (Seattle, WA) and distributed via e-mail to AAN member neurology and child neurology/neurodevelopmental disabilities residents with training ending in 2022. To increase survey yield, 2 reminders were sent, each 1 month apart. Trending comparisons on consistent questions between 2017 and 2022 were conducted using chi-square tests.

Overall, 88 of 985 graduates (9%) responded, fewer than in the previous survey (26%). No significant difference existed between respondents and the nonrespondent 2022 residency cohort for age, self-identified sex, or race (Table). Other demographic information was not available. 98% planned to pursue a fellowship. General neurology, stroke, and neurohospitalist were the most popular career trajectories, each significantly increasing from 2017 (*p* < 0.05). From previous surveys, there was no difference in median student loan debt ($192,500 vs $155,000) (*p* > 0.05), and it continued to moderately influence career choice (mean rating 3.4 vs 4.3 on 0–10-point Likert scale) (*p* > 0.05). 53% of respondents had no business training during residency; only 2% felt very prepared to perform business management tasks, a significant drop from 18% in 2017 (*p* = 0.036). Most respondents with formal or informal experience received training from attendings (90%), a significant increase from 53% in 2017 (*p* < 0.001). Respondents identified unawareness of available resources (92%) and/or lack of time (26%) as reasons for not using business management training resources. 71% of respondents reported dedicated research time. Administrative/clinical duties were the top barrier to conducting research during residency (82%), followed by inadequate training in research skills (40%). 47% felt that they had sufficient outpatient exposure before fellowship decision, yet 79% felt that the fellowship application time line was too early.

**Table:** Demographic Information on Survey Respondents and Nonrespondents Analyzed From the AAN Internal Membership Database

	Respondents (n = 89)	Nonrespondents (n = 894)	Significance testing
Age (mean)	34 (SD = 3.5)	33 (SD = 3.3)	*p* = 0.087
Sex (%)			
Woman	43 (48)	448 (50)	*p* = 0.587
Man	46 (52)	448 (50)	
Race (%)			*p* = 0.872
American Indian/Alaska Native	0 (0)	1 (0)	
Asian	12 (29)	117 (32)	
Black or African American	2 (5)	27 (7)	
White	25 (60)	179 (49)	
Hispanic or Latino	5 (12)	47 (13)	
Other	1 (2)	12 (3)	

There were no significant differences between respondents and nonrespondents. Means were compared using an independent *t* test, and race and sex were compared using a χ^2^ test.

Although the response rate was low, several targets for future investigation and areas of improvement were identified, noting that the period that this survey encompassed included the COVID pandemic, a major limitation. Despite most respondents planning on pursing fellowship training, a large proportion of respondents intend to have a component of general neurology or neurohospitalist in their clinical practice. However, the proportion of graduates who intended to practice only general neurology remained stable. This pattern may highlight a deficiency in residency training programs. Besides interest in a particular subspecialty, this may imply that residents do not feel adequately prepared to practice general neurology after graduation. More respondents in the present survey, compared with the 2017 iteration, indicated interest in neurohospitalist or vascular neurology. The reason for this likely is multifactorial, reflecting a combination of the inpatient-heavy training paradigm of neurology residency around the fellowship application time line, the quality of ambulatory education, and the ever-increasing electronic medical record burden, such as messaging portals. Because the fellowship application time line has shifted later and largely to a match in most subspecialties since the administration of this survey, future iterations should evaluate the effect of this shift.

Similar to 2017, almost half of the respondents reported having student loan debt, which continues to influence career choice. However, the reason individuals choose a career path is multifactorial. Further studies should explore factors contributing to career choices.

The percentage of residents who believe that residency adequately prepared them for specific business and practice management tasks remained consistently low across most areas. The level of underpreparedness in this domain underscores the critical need for residency programs and the AAN to offer and advertise comprehensive business management and practice training. Presently, there is a paucity of work on how to improve or innovate business and practice management education, representing a key opportunity for future exploration.

The duration of dedicated research time varied widely, although most respondents were satisfied with their total time. Barriers to research included administrative/clinical duties and inadequate training in research skills (i.e., methodology and biostatistics). This highlights an opportunity for the AAN to supplement clinical education through developing research-related resources.

Several areas of inquiry for the next survey were identified, including (1) asking about the demographics of training programs and respondents to elucidate a granular understanding of the needs of different program types and (2) determining the reason for sustained feeling of unpreparedness for business management tasks.

As the AAN will continue this endeavor, a crucial goal for future surveys is to significantly increase response rate and robustness of data obtained, a major limitation of the present survey. The AAN should consider setting a threshold for a minimum number of respondents before considering the survey completed instead of using a predetermined time frame. Having a preset, statistically determined response number can guide the frequency of reminder requests and improve the validity of data obtained. The AAN can also consider using incentives (i.e., AAN merchandise or Annual Meeting discounts), to increase response rates. They can also leverage program directors or coordinators, requesting that they advertise the survey to their residents. Finally, there should be physicians and AAN staff from this survey workgroup in the next survey iteration's workgroup to allow for continuity, offering wisdom from what was learned from this administration.

The 2022 AAN Resident Survey revealed multiple unmet needs and areas of improvement. Longitudinally surveying residents is a crucial step in understanding the educational needs of trainees in the rapidly evolving field of neurology and dictate future efforts by the AAN in their unfailing support of neurology trainees.
